# Restoring a Musician's Career: The Use of AlloDerm and a Local Advancement Flap in Lip Augmentation

**Published:** 2020-03-03

**Authors:** James Lee, Elisa K. Atamian, Christopher R. Babycos

**Affiliations:** ^a^Division of Plastic Surgery, Department of Surgery, Tulane University School of Medicine, New Orleans, La; ^b^Division of Plastic Surgery, Department of Surgery, Ochsner Clinic Foundation, New Orleans, La

**Keywords:** lip, augmentation, advancement flap, dermal allograft, hyaluronic acid

## CASE DESCRIPTION

**Figure 1:**
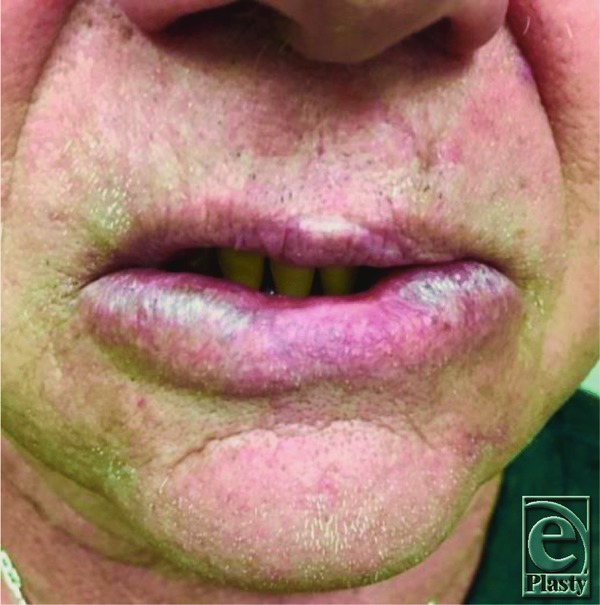
Lip defect on initial presentation.

A 68-year-old professional trumpet player initially presented to us 4 months after undergoing Moh's resection for squamous cell carcinoma of the lower lip, reconstructed with a mucosal advancement flap. The final pathology margins were negative, and both the patient and the surgeon were satisfied with the aesthetic result. His speech and masticatory function were unaffected, but he was unable to play his trumpet, as the soft-tissue depression at the site of reconstruction prevented him from obtaining an adequate seal to his trumpet mouthpiece (Fig 1). Two prior attempts of soft-tissue restoration with hyaluronic acid (HA) fillers were unsuccessful. While the lip defect remained soft and mobile, the HA filler would extrude laterally outside the defect upon direct injection.

On our evaluation, it was evident that the issue was his inability to create an adequate seal on the mouthpiece due to air leakage from the central depression of his lower lip. The patient requested surgical intervention in hopes that he would be able to resume his career as a full-time musician.

**Figure 2:**
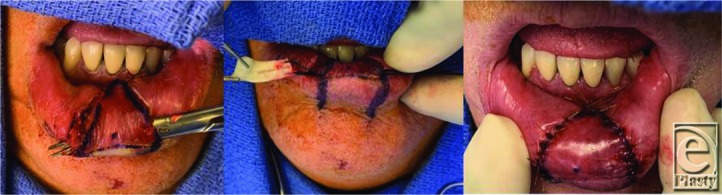
V-Y advancement and AlloDerm inset with closure.

Preoperative markings were made with the patient awake and cooperating with animating his lower lip in the preoperative area. The defect was 2 cm wide and 1.5 cm toward the sulcus. After general anesthesia and intubation, a mucosal V-Y advancement flap was designed. The flap was based on the underlying subcutaneous tissue for blood supply and was advanced 1.5 cm by undermining only the anterior deficient area and advancing the rest of the flap anteriorly. A piece of thick AlloDerm was cut to 25 x 7 mm and tunneled directly into the anteriorly undermined portion of the advancement flap. It was secured into place within the pocket, and the rest of the flap was advanced and closed in V-Y fashion (Fig 2).

## QUESTIONS

1. What are reconstructive options in lip reconstruction?
2. What are the main goals of lip reconstruction?
3. What is the role of allograft in lip reconstruction?
4. Which initial reconstructive option would have been the best choice for this brass player?

## DISCUSSION

Lip squamous cell carcinoma is common in the United States, accounting for approximately a third of oral cavity cancers.^1^ Although it carries a high survival rate, it remains a sensitive area for reconstruction due to its visibility and necessity in basic daily functions. There are a myriad of options available to a patient and the plastic surgeon when approaching lip augmentation. Despite the presenting concern, the goal of lip augmentation should be to yield lasting, functional, and aesthetically pleasing results. Most lip reconstruction techniques after local resection focus on aesthetic and functional restoration by aligning the vermilion border and restoring continuity of the orbicularis oris. While this approach will usually give an acceptable aesthetic result and restore basic sphincter function for speech and mastication, reconstruction in a professional trumpet player presents a complex challenge. In this case, this 68-year-old man was unfortunately unable to work as a musician after his initial surgery. Given his profession, perhaps a better option would have been to reconstruct with more invasive techniques such as wedge excision with primary closure or placement of AlloDerm at the initial surgery.

Acellular dermal allografts have been used for a wide range of reconstructive purposes due to their ability to be effectively revascularized and integrated into surrounding tissues, creating bulk and a new plane of tissue with lasting benefit. The use of AlloDerm in soft-tissue augmentation was first described in the 1990s.^2^^-^4 It has also been used in the lip for aesthetic volume augmentation, with literature demonstrating sustained volume at 1-year follow-up.^4^^,^^5^

AlloDerm is a nonimmunogenic acellular dermal allograft. As a result of its nonimmunogenic nature, patients do not exhibit an inflammatory response to placement of the graft.^6^ This is reflected by reports of low complication rates and overall patient satisfaction after lip augmentation with AlloDerm.^7^ In addition, previous studies have shown low resorption rates of the AlloDerm graft up to 1 year after placement.^3^^,^^6^^,^^8^

**Figure 3:**
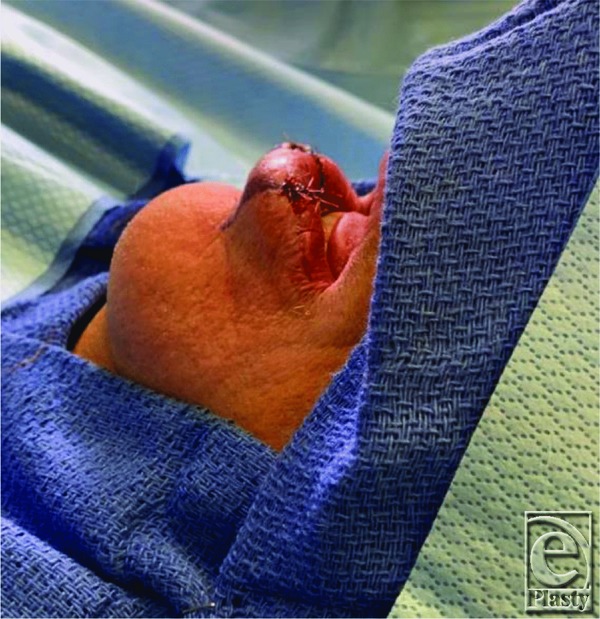
Immediate postoperative result.

**Figure 4:**
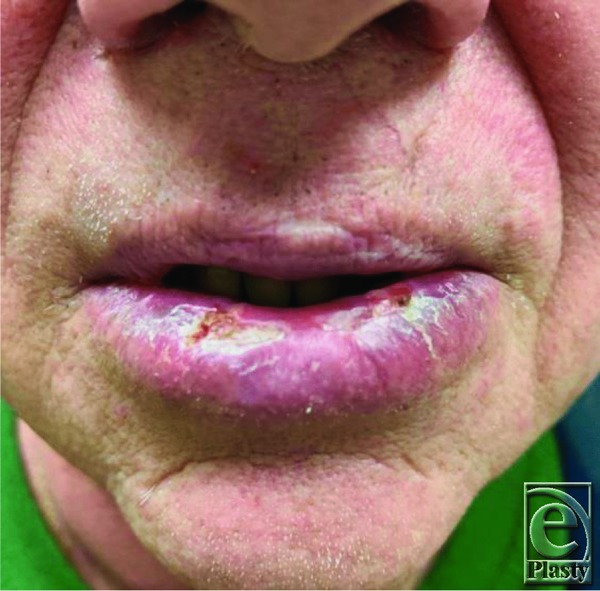
Eight-week postoperative result.

**Figure 5:**
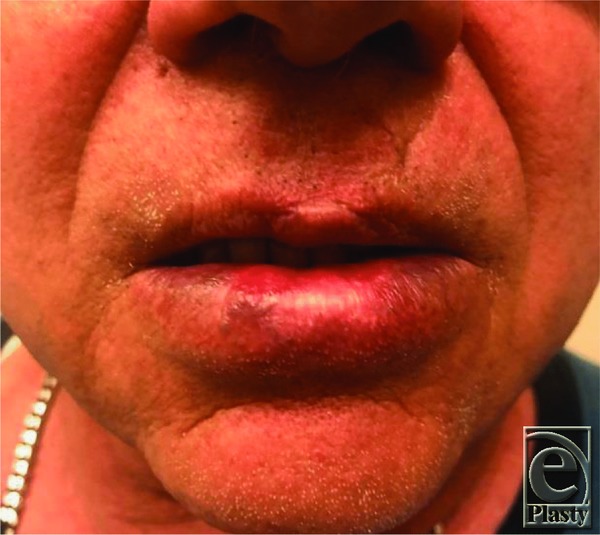
Four-month postoperative result.

By the time our patient presented to the clinic, his lower lip was soft and mobile, as 4 months had already passed. We believe that the initial attempts to “fill” the defect with HA were unsuccessful due to the lack of permanent tissue between the mucosa and the orbicularis oris. The implantation of acellular dermal matrix was performed for this reason, as well as for general bulking of the defect. The V-Y advancement was effective for thickening the mucosal layer already in place by recruiting intraoral mucosa (Fig 3).^9^^,^^10^ The patient was followed up serially for a total of 6 months. All incisions healed without any issues, and he had a good cosmetic result with visual filling of his prior lower lip depression (Fig 4). He was cleared to resume playing at 8 weeks, and his ability to play trumpet was regained, with the exception of high-pitch notes. After visual analysis of his lips while playing the trumpet, we determined that additional bulk was necessary for him to create a better seal. One milliliter of Juvederm Ultra XC was injected into the defect and his ability to play high-pitched notes was improved (Fig 5). He is still able to play the trumpet to this day and is satisfied with his result.

To the best of our knowledge, this is the first report describing the use of acellular dermal matrix along with a local advancement flap in the lip for purposes of reconstructing a segmental soft-tissue defect, with the goal of restoring an air-tight seal upon mouth closure. We believe that this is a viable technique to preserve playing ability in a brass player needing lip excision and reconstruction.

## SUMMARY

The use of acellular dermal allograft has been previously reported in the literature for the use of aesthetic lip augmentation but not for functional reconstructive purposes of the lip. We report a case of a 68-year-old professional trumpet player who was no longer able to play the trumpet after resection and closure of his lower lip for squamous cell carcinoma. He underwent reconstruction with a V-Y advancement flap as well as an acellular dermal allograft to fill in his soft-tissue defect, and his ability to play trumpet was restored.
